# A metamodel-based flexible insulin therapy for type 1 diabetes patients subjected to aerobic physical activity

**DOI:** 10.1038/s41598-022-11772-x

**Published:** 2022-05-16

**Authors:** Emeric Scharbarg, Joachim Greck, Eric Le Carpentier, Lucy Chaillous, Claude H. Moog

**Affiliations:** 1 Nantes Université, École Centrale Nantes, CNRS, LS2N, UMR 6004, Nantes, F-44000 France; 2 Nantes Université, CHU Nantes, Department of Endocrinology, l’Institut du Thorax, Nantes, F-44000 France

**Keywords:** Type 1 diabetes, Biomedical engineering

## Abstract

Patients with type 1 diabetes are subject to exogenous insulin injections, whether manually or through (semi)automated insulin pumps. Basic knowledge of the patient’s characteristics and flexible insulin therapy (FIT) parameters are then needed. Specifically, artificial pancreas-like closed-loop insulin delivery systems are some of the most promising devices for substituting for endogenous insulin secretion in type 1 diabetes patients. However, these devices require self-reported information such as carbohydrates or physical activity from the patient, introducing potential miscalculations and delays that can have life-threatening consequences. Here, we display a metamodel for glucose-insulin dynamics that is subject to carbohydrate ingestion and aerobic physical activity. This metamodel incorporates major existing knowledge-based models. We derive comprehensive and universal definitions of the underlying FIT parameters to form an insulin sensitivity factor (*ISF*). In addition, the relevance of physical activity modelling is assessed, and the FIT is updated to take physical exercise into account. Specifically, we cope with physical activity by using heart rate sensors (watches) with a fully automated closed insulin loop, aiming to maximize the time spent in the glycaemic range (75.5% in the range and 1.3% below the range for hypoglycaemia on a virtual patient simulator).These mathematical parameter definitions are interesting on their own, may be new tools for assessing mathematical models and can ultimately be used in closed-loop artificial pancreas algorithms or to extend distinguished FIT.

## Introduction

Type 1 diabetes is a chronic disease characterized by the autoimmune destruction of pancreatic beta cells, which are responsible for insulin secretion. Artificial pancreas systems have started to become widely used by a new population of patients with type 1 diabetes to mimic natural insulin production^1,2^. Other processes, such as glucose-responsive insulin patches, have been investigated to mimic pancreatic endocrine functions. Curative treatments also form an active field of research, with techniques such as pancreatic islet transplantation and stem cell-based therapies^3^. The majority of artificial pancreas systems are monohormonal, meaning that they utilize insulin only as a control input, but the use of bihormonal devices, including glucagon, as new control inputs has been under investigation for the last decade. A bihormonal therapy enables the active control of glycaemia whether it needs to be increased (glucagon) or lowered (insulin). Due to the presence of these two antagonistic hormones, in principle, it becomes easier to control glycaemia and to prevent hypoglycaemia. Nevertheless, this method comes with limitations as glucagon is a less stable molecule than insulin^4^. Thus, the bihormonal therapy is ignored herein.

Flexible insulin therapy (FIT)^5^ is the methodology that is accepted worldwide for the monohormonal treatment of type 1 diabetes. The treatment process is personalized according to sensitivity factors and ratios that are specific to each individual. These parameters are also related to the parameters of any knowledge-based mathematical model of glucose-insulin dynamics. Note that alternative models, such as models derived from deep learning, are less transparent with respect to those FIT parameters. Models describing of the effects of ingested carbohydrates have been widely worked out. Their management is well understood, and most studies conclude that meal announcement is required to guarantee the performance of a closed-loop system. With regard to taking into account physical activity, the situation is quite different as explained below.

Patients with type 1 diabetes should practise regular physical activity, as it reduces the risk of developing cardiovascular diseases and significantly decreases their insulin requirements^6^. However, this is a difficult situation to manage because patients with type 1 diabetes usually need to decrease insulin to prevent hypoglycaemia.

At a cellular scale, aerobic exercise uses oxidative processes (which require oxygen) to synthesize adenosine triphosphate (ATP) from glucose and thus deliver energy to the organism. These procedures require a constant flow of oxygen to be delivered to the body cells; this requirement, can only be satisfied if exercise remains moderate in intensity. Anaerobic refers to exercises corresponding to high-intensity physical activities (weightlifting, sprinting,etc.) and can paradoxically lead to hyperglycaemia. Aerobic physical activity (walking, running, swimming, cycling, etc.) is known to be hypoglycaemic and is the main type of physical activity practised by the average population. Both types of physical activity are energy-consuming and therefore require glucose to be transported to muscle cells to synthesize ATP. GLUT-4 glucose transporters fulfil this role. These transporters are mainly distributed in striated skeletal muscle cells as well as in cardiac muscles and adipose tissues where they allow glucose to be transported into cells. The translocation process is either insulin-stimulated or contraction-induced. Three main biological mechanisms lead to increased glucose uptake in muscle tissues: (1) an increase in local muscular blood flow is responsible for more insulin being driven towards muscle tissues and thus leads to a higher glucose intake level due to insulin-stimulated GLUT-4 whose capacity is reduced but not annihilated in patients with diabetes; (2) enhanced insulin receptor (IR) sensitivity^7^ ; and (3) an activation of contraction-induced GLUT-4 due to exertion.

At this stage, a set of open problems can be listed. Although the knowledge of the FIT parameters is crucial for medical practitioners, these parameters are usually omitted by the scientific community when deriving new models for insulin-glucose dynamics in type 1 diabetes cases. Well-defined FIT parameters would help to assess mathematical models and would ultimately enable better control over two major disturbances: meals and physical activity.

As explained above, models that take physical activity into account are still works in progress. Today, we have an opportunity to detect physical activity due to measured activity variables (heart rate, actimetry, etc.)

In the case of diabetes, exogenous insulin does not adapt in a physiological manner once it has been administered, which can lead to hypoglycaemia. Thus, better glycaemic control, even during exertion, is one of the main drawbacks to overcome, as it prevents patients with type 1 diabetes from regularly exercising due to the fear of hypoglycaemia^6^. Multiple precautions must be taken to prevent hypoglycaemia, i.e. physical activity should be started close to hyperglycaemia (approximately 1.8 g/l) and the basal insulin flow must be reduced before and during activity.

Our findings aim to provide solutions to the above open problems.

A metamodel for glucose-insulin dynamics that is, subject to carbohydrate ingestion and aerobic physical activity is derived; it incorporates major existing knowledge-based models. The common core, Bergman’s minimal model, is highlighted. Our own new model is challenged with clinical data and in silico testing.

We derive a comprehensive and universal definition of the underlying FIT parameters as the insulin sensitivity factor (*ISF*). Our approach allows us to extend and specialize these notions to any model regardless of its complexity. In addition, the relevance of physical activity modelling is assessed on clinical data.

Ultimately, the list of FIT parameters is enriched to include physical exercise. This involves an *extended* FIT which yields a new control law to be applied. Its feasibility is sound as it is based on new sensor readings as heart frequency, or actimeters. We can close the loop by taking real-time physical activity into account, thereby providing a fully automated closed insulin loop while maintaining the best performance available. The corrections in the insulin infusion rate are updated with respect to the standard FIT. Specifically, we cope with physical activity by using sensors leading to the maximization of the time spent in the glycaemic range (76% of time in the range from 70 to 180 mg/dl and 1.3% of hypoglycaemic time on a virtual patient simulator).

## Results

### The metamodel based Flexible Insulin Therapy parameters

**Figure 1:**
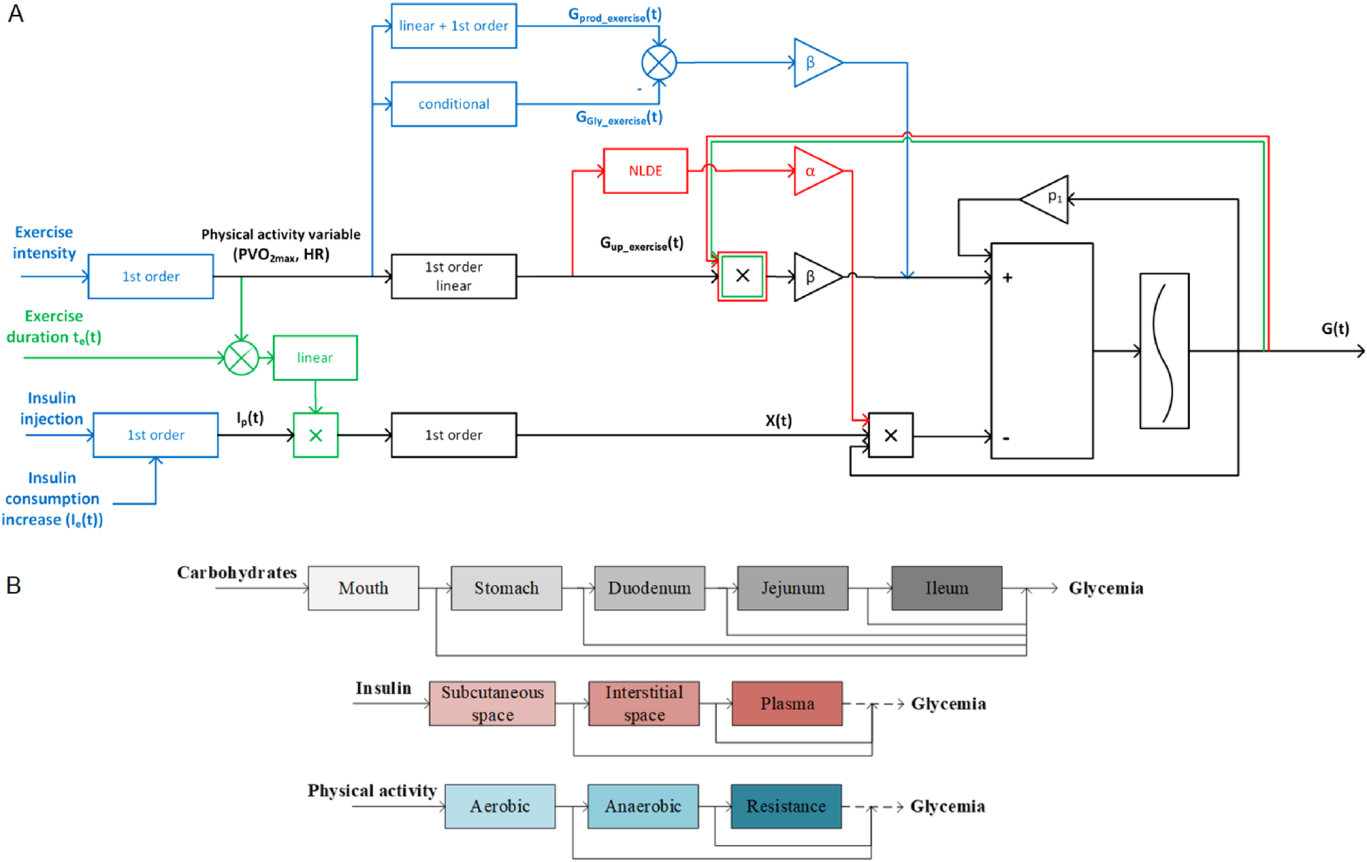
Metamodel of the glucose dynamics subject to insulin injection and physical activity. (**A**) Top: A metamodel based on the Bergman minimal model. The black bold scheme represents the core that is common to three specific models, namely Roy and Parker^8^ in blue, Breton^9^ in red and Alkhateeb^10^ in green. The most exhaustive model in terms of biological processes considers the factthat physical activity increases hepatic glucose production, also causes a depletion of glycogen stocks leading to a decrease in the glycogenolysis rate and finally increasing the glucose uptake of working tissues. Other models synthesize these three mechanisms in one module by considering only the increase in the glucose uptake. The ingested carbohydrates can be easily added to these schemes as a third input channel. (**B**) Bottom: A multicompartment metamodel for the diffusion of carbohydrates, insulin and physical activity and their effects on glycaemia. These multiple compartments act as a ’buffer’ for the diffusion of the primary input. Their behaviours are described in mathematical diffusion equations, which are linear in the case of injected insulin diffusion or in the case of carbohydrates digestion. However, they are nonlinear for some physical activity models.

Figure 1 displays a metamodel that is focussed on physical activity and three models that are available in the current literature. Further disturbance input channels may be added to complete the scheme. A comprehensive and universal definition of the sensitivity factors (to external inputs) is given next.

FIT aims at personalizing the treatment provided to each individual patient based on sensitivity factors that enable the quantification of specific exogenous insulin needs in response to specific external disturbance inputs (i.e., carbohydrates, stress, or physical activity). This medical approach is the cornerstone of our work, as we aim to capture real-life clinical practice. To this end, we design a new mathematical model derived from the so-called FIT model, or the Minimal Model Control-oriented^11^, including the hypoglycaemic action of aerobic physical activity. A new physical activity sensitivity factor is introduced and shown to be instrumental to tune the amount of injected insulin according to the type and intensity of physical activity performed.

Physical activity models from the literature^8–10,12^ are merged into a single metamodel, which is sketched in Fig. 1. Notably, the various contributions are not consistent in terms of the biological processes to be modelled and specifically in regard to the definitions of sensitivity factors.

This metamodel in Fig. 1 is completed by the following mathematical metamodel which includes major continuous time models that are available in the literature. By slightly abusing the notation, we write:

1 $$\begin{aligned} \left\{ \begin{array}{rcl} {\dot{G}}&{}= &{}{\dot{G}}(G,X_i^1,...,X_i^p,u_i,...,X_c^1,...,X_c^q,u_c) \\ {\dot{X}}_i&{}= &{} {\dot{X}}_i(X_i,u_i)\\ {\dot{X}}_c&{}= &{} {\dot{X}}_c(X_c,u_c) \end{array} \right. \end{aligned}$$

where *G* denotes the blood plasma glucose concentration, $X_i^j$ denotes the *p* intermediate insulin compartments and $X_c^k$ represents the *q* intermediate carbohydrate digestion compartments. The notations $X_i$ and $X_c$ stand for the vectors $X_i=(X_i^1,...,X_i^p)$ and $X_c=(X_c^1,...,X_c^q)$, respectively. When physical activity is involved, a new subsystem is added with a variable $X_{HR}$ and driven by the heart rate $u_{HR}$, which is considered an external input. Further developments are found in the Supplementary Information (Section 1). Figure 1 displays the metamodel built around a common core which is the Bergman minimal model^13^. Therein, R. Bergman gave the first mathematical definitions of glucose Effectiveness *E* and insulin sensitivity *ISF*. ’Glucose effectiveness’ refers to the ability of glucose to suppress endogenous glucose production and stimulate glucose uptake. It is computed as $E=-\frac{\partial {\dot{G}}}{\partial G}$.

In medical terms, insulin sensitivity is a factor that mitigates the decrease in glycaemia caused by the injection of one unit of insulin. For the Bergman model, it is the insulin capacity required to lower the blood glucose concentration and is expressed as $ISF=-\frac{\partial ^2 {\dot{G}}}{\partial I \partial G}$ under steady-state conditions. The latter is only valid for a Bergman-like model structure that consists of a cascade of two insulin compartments and which introduces insulin-dependent glucose uptake as a bilinear term between *G*(*t*) and *X*(*t*), some intermediate insulin compartment.

With mathematical models becoming more complex, including several remote insulin compartments as shown in Fig. 1B, the above formula for the *ISF* is no longer valid. A generic definition that is able to cope with (1), and thus is usable with almost any mathematical model is crucially needed. Analogous sensitivity factors with respect to any type of external disturbance input are worth defining accurately, whether the disturbance is embodied by the ingested carbohydrates $u_c$ by some physical activity $u_{HR}$ or any other external input.

In the special case of carbohydrates, it is quite obvious that meals with different glycaemic indices have their glucids assimilated by blood plasma at different digestion compartments. The model structure shown in Fig. 1B is relevant.

When defining a sensitivity factor, we consider the relative variation in the output ${\dot{G}}$ with respect to an external input, (or eventually with respect to the glucose concentration to capture glucose effectiveness as well). This general formulation allows us to consider not only the injected or ingested quantities but also the amounts of each that, are still active in intermediate compartments. The insulin sensitivity factor derived from the metamodel becomes

2 $$\begin{aligned} ISF=d{\dot{G}} \cdot \left( \begin{array}{c}0\\ k^d_{1i}\\ \vdots \\ k^d_{pi}\\ 1\\ 0\\ \vdots \\ 0 \end{array} \right) \end{aligned}$$

where $d{\dot{G}}$ is the differential (or gradient) of ${\dot{G}}$ and the column vector consists of the static or instantaneous gains of the *p* insulin diffusion compartments. This definition takes not only the explicit inputs on the right-hand side of ${\dot{G}}$, but also the total quantity contained in all diffusion compartments into account.

Further details are found in the Supplementary Information of this article, which includes the formal definitions of the sensitivity factors. They are computed in the special case of some renowned models according to (2). Applying this definition to the new FIT model (or extended FIT model) introduced in this paper yields an *ISF* expressed in [mg/dl/U].

The methodology is exactly the same for every sensitivity factor with respect to any other input, taking the static diffusion subsystem gains of interest into account. In the same way, the new physical activity sensitivity factor *PSF* is defined in terms of the static or instantaneous gains of the *q* digestion compartments:

3 $$\begin{aligned} PSF=d{\dot{G}} \cdot \left( \begin{array}{c}0\\ 0\\ \vdots \\ 0 \\ k^d_{1c}\\ \vdots \\ k^d_{qc}\\ 1 \end{array} \right) \end{aligned}$$

The *PSF* is expressed in [mg/dl/bpm/min] and characterizes the glycaemia decrease induced by exertion. A physical activity to insulin ratio (*PIR*) results from the ratio between the *ISF* and the *PSF*. The *PIR* is expressed in [bpm/U]. This ratio is used in the design of a new control law to compute the units of insulin to be removed from the injected insulin due to physical activity.

The main findings at this stage are summarized as follows. Whatever the metamodel consists of, whatever the model is, and whatever the control inputs or disturbance inputs of interest are, the sensitivity factor w.r.t a specific input is the scalar product of the gradient of $\dot{G}$ and the static gains vector (of all compartments) w.r.t. this input.

Now, we are ready to consider any model and any input (stress, external temperature, any coinfection...) to characterize the associated sensitivity factors. This general definition remains valid for general structures of the metamodel, including couplings in the diffusion subsystems. This result is of technical interest.

### In silico assessment of the new model and associated control

**Figure 2:**
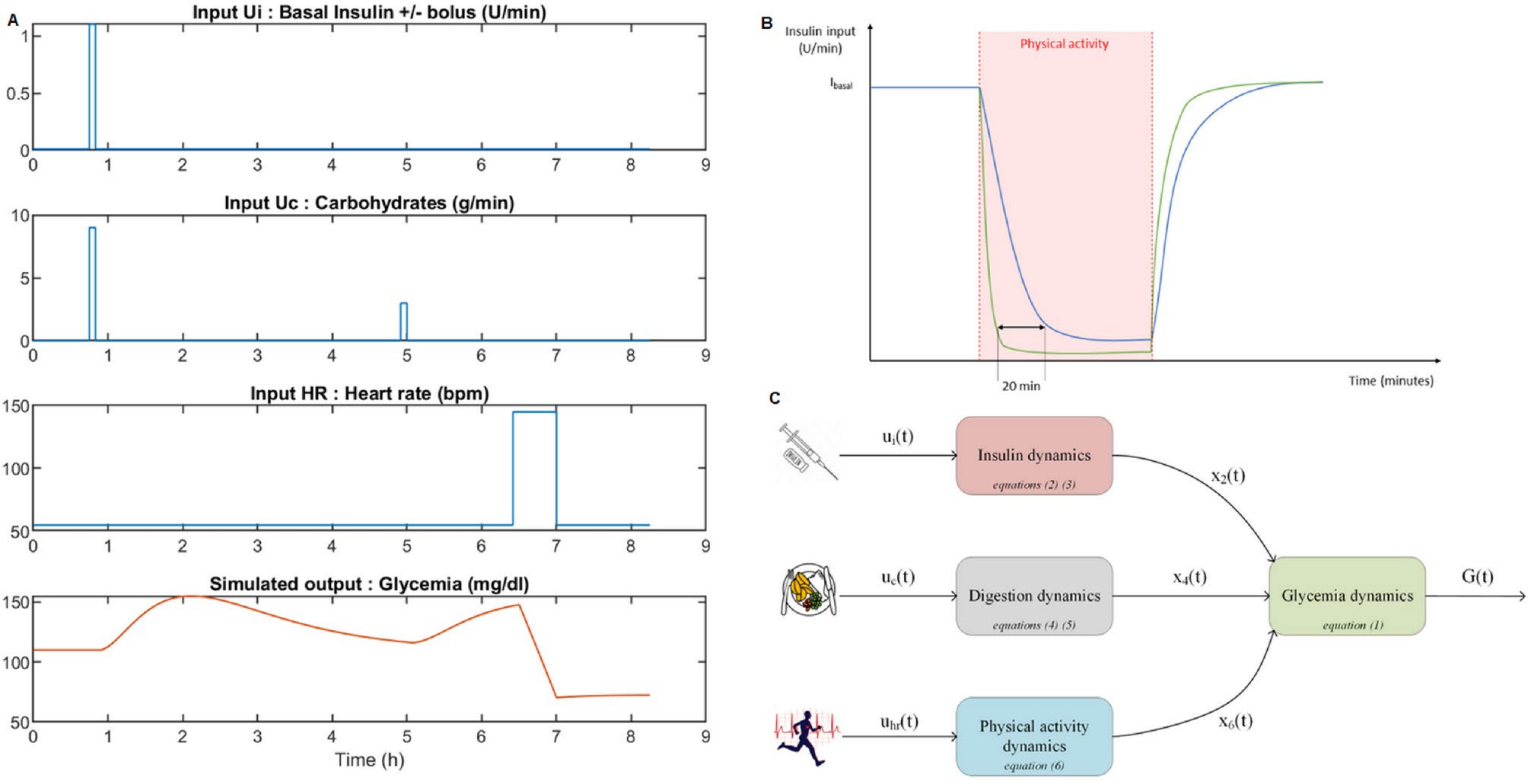
The extended FIT model and control law behaviour in the simulation. (**A**) Simulation scenario used to assess the extended FIT model behaviour in reaction to different disturbances. $U_i$ stands for the insulin input flow and is in [U/min], $U_c$ corresponds to the ingested carbohydrates’ flow in [g/min] and $U_{hr}$ is the heart rate in [beats/min] or [bpm]. (**B**) Diagram highlighting the benefits of a control law with a physical activity variable (in green) and with only a glycaemic regulation term (in blue). In response to aerobic activity, the use of data such as heart rate can enable a quicker reactions to glycaemia decreases. (**C**) The extended FIT model structure, each block represents a subsystem of differential equations.

The extended FIT model consists of 6 ordinary differential equations with four main dynamics, as shown in Fig. 2C. Insulin diffusion dynamics are modelled with a two-compartment subsystem including: a subcutaneous compartment and a blood plasma compartment. A two-compartment model is also chosen to model the digestion subsystem, including the stomach and the duodenum. These second order models are consistent with the pharmacokinetics of insulin and the appearance rate of glucose in blood plasma after a meal. Finally, physical activity is assumed to have a delayed impact on blood glucose concentration; therefore, additional first-order dynamics, which are driven by the heart rate input and define a physical activity variable, are introduced. The mathematical term describing the impact of physical activity on blood glucose synthesizes three biomechanisms that occur during exertion in type 1 diabetes patients. Exogenous insulin is unable to physiologically adapt to physical activity. Muscular tissue increases its glucose consumption and a counter-regulation action is inhibited by excessive insulin levels in the circulatory system. This is summarized in one unique additional term in the glycaemia dynamics model, with a hypoglycaemic effect (see Supplementary Information).

The behaviour of the extended FIT model (4) is first assessed with simulations in Fig. 2A. This model is restricted to the case of aerobic exercise as this is the main type of physical activity performed within our cohort of patients. The test scenario is initialized with a glycaemia level that is within the homeostasis target with a well tuned basal rate. This means that the blood glucose level remains constant at 110 mg/dl. A meal situation is evaluated with an adequate number of concomitant insulin bolus units. Before a physical exertion, each patient ingests 15 g of carbohydrates without any insulin injection prior to starting physical activity in an acceptable glycaemic range. Then, a medium intensity exercise lasts for 30 min and causes glyacemia to decrease.

A control law is now computed from the previous model. Its goal is to maintain glycaemia around a reference value while compensating for meals and physical activity. Heart rate readings are used to detect physical activity and enable a faster response for the control. In Fig. 2B, we compare the responses of two control laws, one that utilizes only a regulation-like process that compares the glycaemia output with a reference value and another that utilizes the heart rate readings. In some cases, especially when the patient’s sensitivity to physical activity is low, the regulation control takes an extra 20 min to become effective, whereas the control process accounting for physical activity lowers the insulin flow in a couple of minutes. The need for a control law that takes physical activity into account becomes even more blatant when modelling a Continuous Glucose Monitor (CGM). Ultimately, including a 15 min delay in the model of the CGM sensor, we are able to mitigate the decrease of glycaemia by approximately $10\%$, with respect to the case where insulin remains at its basal level during exertion. In nondeclarative control algorithms, such as the one developed herein, it is imperative to detect exertion as soon as possible (as in real-life conditions). A patient is advised to lower his basal insulin rate at least 20 min before performing exercise.

The control law is intended to perform well as long as the patient prepares himself/herself appropriately. For example, if the patient starts exercising with low glycaemia or without any prior carbohydrate intake^14^. The control law will be able to slow down the decrease in blood glucose concentration but will not be able to reverse the trend as long as exercise continues. The ultimate goal is to guarantee more security regarding hypoglycaemia and enable longer physical activity periods.

### Model parameter identification from clinical data

**Figure 3:**
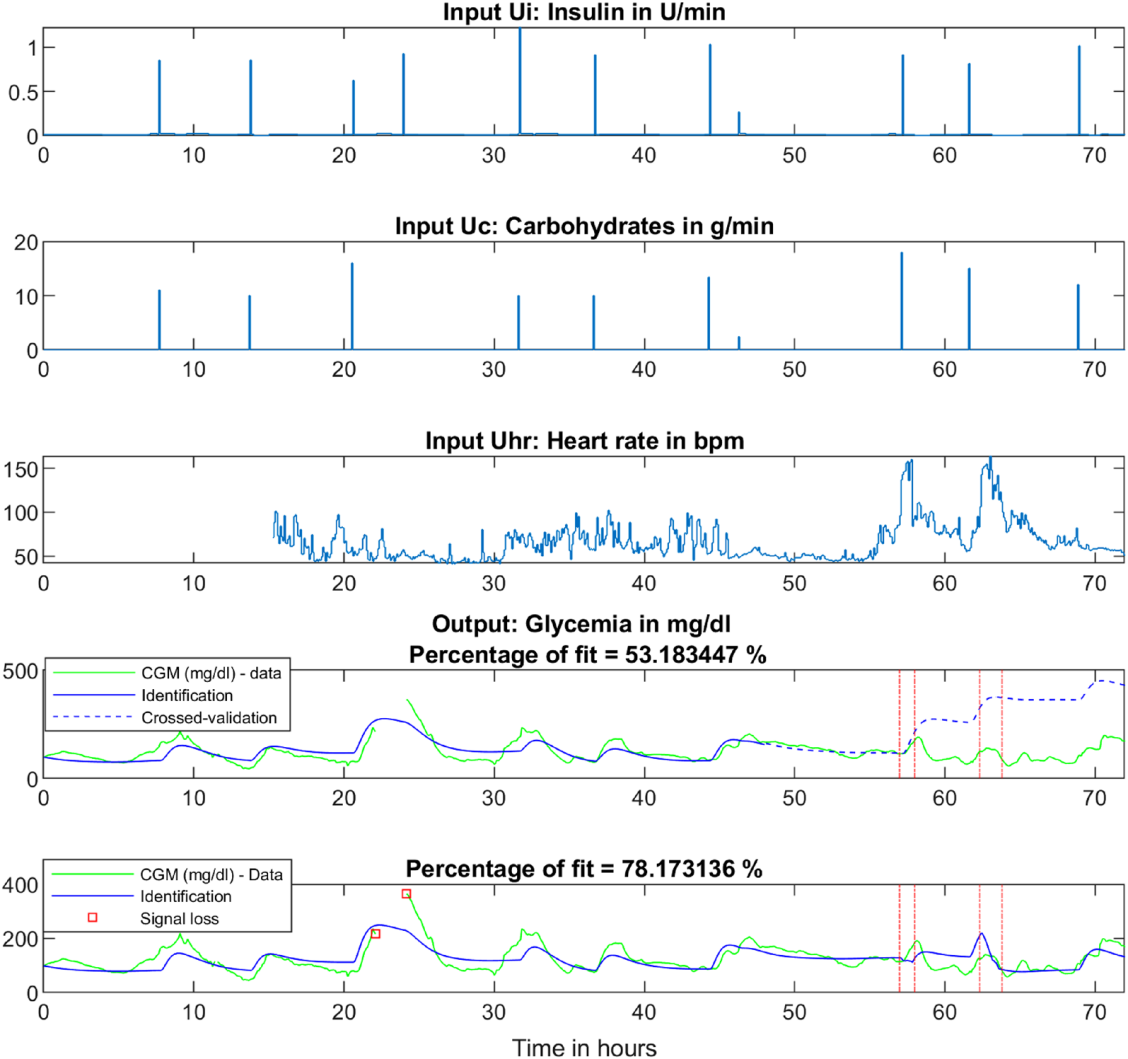
The identification procedure led by clinical data with a physical activity period. The upper panel represents the insulin input in [U/min]. These input data are retrieved from the patient’s insulin pump. The second panel pictures meal intakes in [g/min] which are declared by the patient. The third graph and last input is the heart rate, which is acquired using a smartwatch with a plethysmograph. During the first 15 h, the heart rate sensor malfunctions, and we assume that the heart rate remains at a reference value of 55 bpm as the actimetry data show no exertion. The fourth panel shows an identification procedure performed over 48 h via a model without physical activity equations followed by a crossed-validation of 24 h, showing that the model fails and quickly diverges after the onset of exertion. The last graph considers the same 72 h of data while conducting identification on the whole data set with the extended *FIT* model (including physical activity). The four red vertical lines the delimit two physical activity periods where exertion is deemed significant.

We perform an identification procedure on clinical data to demonstrate the fitting performance of our model (Fig. 3).

After defining a new mathematical model, we verify that accounting for physical activity is essential for capturing the insulin-glucose dynamics of an individual. An identification procedure is implemented on clinical data obtained under free-living conditions to answer this first question. The patient of interest is a 50 year-old male practising for a triathlon. Physical activity is detected when the aerobic threshold (approximately 50% of VO$_{2max}$) is reached.

Identification is used to validate modelling assumptions and assess the different models that are available in the current literature. We test multiple configurations with either nonlinear terms or parameters designed to represent the long-term effect of physical activity on the *ISF*. It is observed that the selected *FIT* model (4), presented in the Methods section, is the optimal choice in terms of performance and simplicity, as its fitting percentages is larger or equal to the fitting percentage of other evaluated models.

By using the sensitivity factor definitions (2, 3) introduced in the Results section, we are able to calculate these factors for the patient of interest. In the specific case of the extended *FIT* model, the *ISF* equals $k_i$. With the identified parameters values, we obtain $ISF=26.05$ mg/dl/U.

The *PSF* is equal to $\beta =0.03$ mg/dl/bpm/min. Finally, the *PIR* is computed as $PIR=ISF/PSF=868$ bpm.min/U. This coefficient is interpreted as a response to a piecewise constant representing physical activity. More precisely, considering a standard physical activity duration of 30 min, the *PIR* is $PIR_{30\,\mathrm{min}}=29$ bpm/U meaning that an increase of reference heart rate of 29 bpm over 30 min will have an effect equivalent to that of an injection of one unit of insulin.

### Control law assessment on virtual patients

Here, we present an in silico test of our control law (8) with an evaluation of the time spent in the glycaemic range for both our virtualized patient data set and the Oregon Health and Science University (OHSU) simulator^15^.

**Figure 4:**
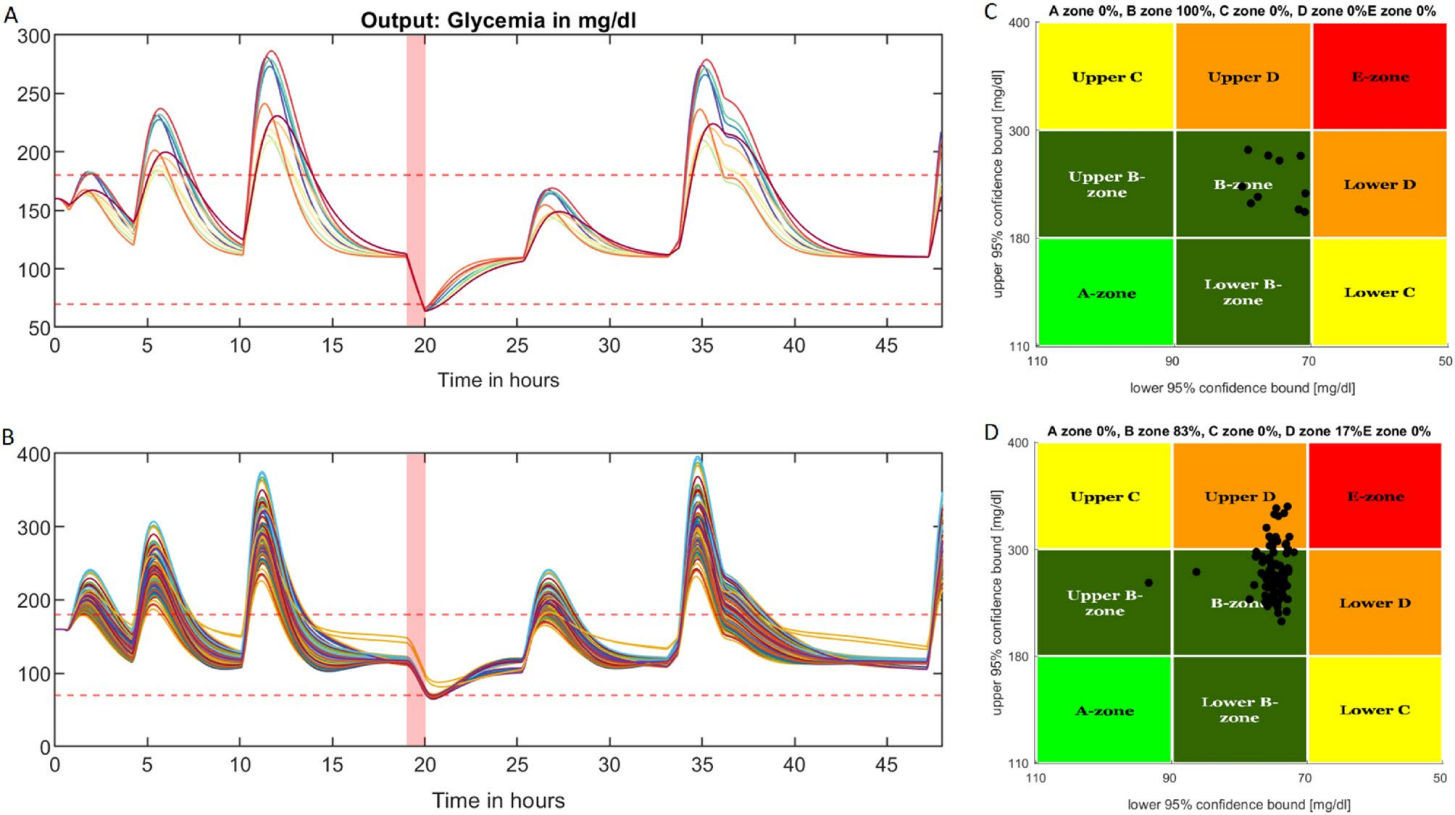
Control law performance comparison on a cohort of virtual patients and a simulator from the literature. (**A**) Simulation scenario and the results of our 10 virtual patients obtained by using our state-feedback controller. The horizontal dashed-lines represent the hypoglycaemia and hyperglycaemia thresholds (resp. 70 mg/dl and 180 mg/dl), respectively. The red-coloured area corresponds to the physical activity period. The 48-h scenario presented here included 3 meals on the first day, followed by an exercise bout and 3 more meals the next day. (**B**) Simulation of the 99-patient cohort from the OHSU simulator using our control algorithm and the same scenario as above. The horizontal dashed-lines have the same meanings as those in panel (**A**). (**C**) Control-variability grid analysis for our patient cohort with each black dot representing a patient according to their time spent in and out of the glycaemic range with a 95% confidence interval. All patients remained in the B-zone meaning that the control law kept glycaemia within the target range. (**D**) Control-variability grid analysis for the OHSU simulator.

After obtaining the first identification results, the control law was tested on our virtualized patient cohort (in the simulator based on our model) which consisted of 10 patients in free-living conditions during a 48-h scenario including 1 h of medium-intensity physical activity. To do so, an identification procedure was perfomed on the 10 patients to find a set of fitting parameters. Then, state-feedback (8) was applied, yielding the graph in Fig. 4A. This figure shows the patient intervariability levels produced with different responses to meal intake and physical activity, along with magnitude changes and time constants; furthermore, the control process behaved consistently for every patient, allowing us to maximize the time spent in the glycaemic range. The mean time spent in the glycaemic range by the 10 virtualized patients was $81.8\%$. The time spent in hyperglycaemia averaged $16.8\%$ and the time spent in hypoglycaemia was evaluated at $1.4\%$.

A similar procedure was performed on the OHSU virtual patients simulator to assess the performance of our control law. This OHSU simulator^15^ provided a cohort of 99 patients under a single-hormone therapy. The default scenario implemented in the simulator was chosen to complete the assessment. This was the same scenario as that used in our 10 patient-cohort. Figure 4B displays the performance of the closed-loop on the virtual patient cohort. The physical activity period started at 7 pm on the first day. A control-variability grid analysis was plotted in Fig. 4C and d for both cohorts to assess the performance of the control law in terms of time in the glycaemic range. Because some meals contain significant amounts of carbohydrates and because the control law only administers insulin once meal starts, glycaemia tends to increase more in this case than with an anticipative bolus injection when the meal is announced in advance. In this scenario, as the patients did not start physical activity at an adequate blood glucose concentration (i.e., approximately 180 mg/dl^16^), the scatter plot tended to shift to the right towards the 70 mg/dl hypoglycaemic limit. Despite these limitations, the control law demonstrated its ability to control glycaemia with a mean time-in-range (*TIR*) percentage (i.e., time spent between 70 and 180 mg/dl) of $75.5\%$ which met recommendation 6.5b^17^ of the American Diabetes Association that at least $70\%$ of time should be spent in the target range for good glycaemic control, and a mean percentage of time spent in hypoglycaemia of $1.3\%$ (recommended: below $4\%$).

## Discussion

Figure 3 shows the necessity of modelling physical activity, as the cross-validated curve was not able to follow the true insulin-glucose dynamics as soon as the first exercise period occured. We could tell from the first activity period that the patient purposely lowered the insulin bolus associated with his meal as the modelled glycaemia in dashed line hardly decreases after the meal; this is contrary to what one might have expected. When identifying the 72 h of data with the extended *FIT* model, we saw that the meal occurring just before the first activity period was well-compensated by physical activity, as was the second meal at approximately 62 h. This result strongly suggests that modelling of physical activity represents a significant improvement for predicting glycaemia and that our model can efficiently describe natural glycaemic variations.

The metamodel introduced above aggregates several models including meal intake and aerobic physical activity, that are available in the literature. From this metamodel, we provide a universal sensitivity factor definition, which represents a crucial step in the modelling of type 1 diabetes, thereby enlightening the hidden outcomes of the actual literature models^8–10,12,13^ and their inherent links with FIT. To date, some models have hardly been related to medical practice; now all the relevant factors can be expressed for every knowledge-based model. The properties of the obtained factors and their consistency with clinical FIT parameter values form a way to assess the validity of a given model. The definition of the *ISF* in (2) represents the sensitivity to the injected exogenous insulin, which is of interest to diabetes practitioners, and not the sensitivity to the blood plasma insulin concentration which is quite common in current studies.

The proposed model (called the extended FIT model) is completely in line with the FIT theory and the metamodel definition; it also contains parameters with consistent units in terms of clinical practice.

From identifications conducted on clinical data, it was unclear that the bilinear term chosen by Breton (as detailed in the Supplementary Information) was the most relevant term for describing the evolution of glucose consumption. Additionally, no biological explanation that could justify a product between the physical activity variable and glycaemia was found. In silico tests did not show evidence that physical activity has a major impact on the *ISF*. This impact has been widely documented and accepted; however, to the best of our knowledge, no study to date has provided any evidence of the importance of modelling such a mechanism. In fact, all bioengineering articles presenting models and their performances either worked on simulated data or worked on real data but focused on small time windows; this does not seem to be appropriate for assessing the importance of so-called “long-term” effects on glycaemia. Notably, the new long-term impact parameter in our model enlarges the applicable family of models and may yield slight improvements when fitting identification results. Thus, to keep the mathematical model as simple as possible, we decided to keep the insulin sensitivity constant during physical activity.

The new extended *FIT* model was validated against clinical data acquired from type 1 diabetes patients without any preprocessing apart from synchronizing the different device clocks. A significant enhanced of fit was achieved when using the model that accounts for aerobic physical activity. It is also important to stress that no CGM device modelling was introduced in this work; consequently, it was assumed that we instantaneously acquired the plasmatic glucose levels. However, it is more realistic to consider that such a control law will more likely be used with an interstitial glucose sensor such as a CGM. As CGM measures obtained during physical activity can lag behind capillary or venous blood glucose measurements by 5 to 28 min^14^, it is likely that glycaemia will be overestimated, leading in our case to an even more delayed control action in terms of reducing the insulin flow and making the use of heart rate or actimetry data even more crucial. This further demonstrates the importance of both the new control law and of accounting for physical activity, which embodies a new disturbance input that can hardly be neglected.

Based on our cohort of 10 patients, virtual patients were designed to develop our own simulator for testing the new control law. Due to communication issues such as CGM signal loss and errors caused either by the smartwatch or by the patient when reporting meals, some patients had their parameters identified with a low accuracy, but good approximations of the clinical parameters were still obtained. For both our virtualized patient cohort and the OHSU simulator, we obtained performances exceeding the recommendations of the American Diabetes Association^17^. Our patients spent a mean of $81.8\%$ of their time in the glycaemic range (70-180 mg/dl) and experienced only 1.4% in hypoglycaemia during a typical day with physical activity under our control law. Resalat et al. achieved 78.1% of time spent in glycaemic target range and 3.4% of the time spent below the target with the OHSU simulator and the control law developed in^15^. As the implemented scenario was the same as that experienced by their patients *in vivo*^15^, the above percentages can be compared to the actual values of 74.3% and 2.8% obtained for the real OHSU patients. The authors acknowledged that their simulator tends to overestimate time-in-target values, whereas our simulator provides more realistic outcomes with less hypoglycaemia.

A major breakthrough of our work concerns the translation of the mathematical control law to an Extension of the Flexible Insulin Therapy (8) providing a new tool for patients and medical practitioners to anticipate aerobic physical activity. Due to the *PIR* which can be evaluated through a standard effort test performed on a treadmill, the patient can calculate their insulin flow rate decrease that must be considered to compensate for physical activity. The *PIR* can also be used to forecast the joint effects of pre-exercise carbohydrate ingestion and physical activity. One can also convert physical activity into units of insulin by using the Physical activity to Insulin Ratio, the *PIR*, to better understand the effects of physical activity on the body and relate it to a known variation in type 1 diabetes patients. Nevertheless, these calculations must be handled with caution, as a global understanding of each input time-response is necessary to act at the right moment. This approach basically formalizes what patients have done this far to prevent hypoglycaemia during sports and strengthens the methodology with some theoretical arguments. The design of a validated insulin-glucose dynamics simulator that takes physical activity into account remains a perspective for a further assessement of our results. The results presented here are theoretical results at this stage, and they deserve to be challenged through a clinical trial.

## Methods

### Mathematical modelling

A mathematical model was developed in this work as an extension of the so-called flexible insulin therapy model^18^, also named the ’Minimal Model Control-oriented’^11^. The latter has demonstrated its long-term ability to reproduce the insulin-glucose dynamics of type 1 diabetes while remaining quite elementary, comprehensive to practitioners, and mathematically consistent in terms of its equilibrium points as well as its *a priori* identifiability^18^ from glycaemia measurements. The series generation approach of the GenSSI MATLAB$^{\copyright }$ toolbox was used to show that the parameters were structurally and globally identifiable. A structural and practical identifiability analysis was performed on the flexible insulin therapy model^18^ by Garcia-Tirado^11^, who discussed arguing potential identification issues due to the low sensitivity of the output to certain model parameters.

The physical activity equation introduced in our model was inspired by Breton’s work in^9^. Nevertheless, some modifications were made in order to make the model fully functional on long-term real-life data. We considered an exertion threshold at which point glycaemia started to fall during physical activity. This is mandatory when utilizing free-living data, as the patient heart rate might increase from the reference value when standing or walking; however, this does not necessarily come with a significant increase in glucose consumption. We chose the aerobic threshold as the limit value to consider noticeable impacts of physical activity on blood glucose concentration. Additionally, we assumed that the increased glucose consumption evolved in a linear fashion with physical activity, as in the work of Roy and Parker^8^ and unlike the work of Breton, who considered a nonlinear term.

The extended flexible insulin therapy model including physical activity consists of six equations, three inputs, namely the insulin injection rate $u_i$ in [U/min], the carbohydrate ingestion rate $u_c$ in [g/min] and the heart rate $u_{HR}$ in [bpm] (beats per minute), and one output which is the blood glucose concentration $x_1$ in [mg/dl]:

4 $$\begin{aligned} {\left\{ \begin{array}{ll} {\dot{x}}_1(t)=-k_i.x_2(t)+k_c.x_4(t)-\beta .x_6(t)+k_d\\ {\dot{x}}_2(t)=-\frac{1}{T_i}.x_2(t)+\frac{1}{T_i}.x_3(t)\\ {\dot{x}}_3(t)=-\frac{1}{T_i}.x_3(t)+\frac{1}{T_i}.u_i(t)\\ {\dot{x}}_4(t)=-\frac{1}{T_c}.x_4(t)+\frac{1}{T_c}.x_5(t)\\ {\dot{x}}_5(t)=-\frac{1}{T_c}.x_5(t)+\frac{1}{T_c}.u_c(t)\\ {\dot{x}}_6(t)=-\frac{1}{\tau _{HR}}.x_6(t)+\frac{1}{\tau _{HR}}.(u_{HR}(t)-HR_b)\\ \end{array}\right. } \end{aligned}$$

where the state variable $x_2$ is the plasma insulin flow in [U/min], $x_3$ is the insulin flow in the subcutaneous compartment in [U/min], $x_4$ represents the carbohydrate flow in the duodenum in [g/min], $x_5$ is the carbohydrate flow in the stomach and $x_6$ denotes the filtered heart frequency in [bpm].

The model parameters include $k_d$ – the difference between the endogenous hepatic glucose production value, and the insulin independent glucose consumption value, which is expressed in [mg/dl/min]. The parameter $k_d$ yields hyperglycaemic behaviours of patients with diabetes when all other inputs are zero. The parameter $k_i$ is the insulin sensitivity factor expressed in [mg/dl/U], $k_c$ is the carbohydrate sensitivity factor in [$dl^{-1}$], and $T_i$ and $T_c$ are time constants in [min]. The latter represent the diffusion time in the insulin compartments and the diffusion time in the digestion compartments, respectively. Parameter $\beta$ is the physical activity sensitivity factor expressed in [mg/dl/bpm/min]. Finally, $\tau _{HR}$ is the response time of the heart rate to the onset of physical activity.

### Clinical data and identification procedure

In the special case without exercise, i.e. $$\beta =0$$ and (4) reduces to its first five equations. The identifiability of this model was demonstrated in previous works^18^$$^,$$^19^. By using the MATLAB GenSSi toolbox, it was shown that (4) fulfilled the multi-experiment identifiability conditions so that the seven parameters could be derived from the measured output, under well chosen scenarios (Fig. 1 in the Supplementary information).

When possible, we found that running the optimization algorithm on a two day-long data-set provided that were consistent with the parameters observed in clinical practice.

The clinical data used in this study were provided by Nantes University Hospital. They were obtained from free-living conditions; the patients had to declare their meals and wear smartwatches that were able to acquire heart rate and actimetry data. The maximum heart rate was determined via an estimate given by Gellish *et al.*, which is a curvilinear definition linking maximum heart rate with age^20^. Ten patients have been included in this study to date and one experienced medium-intensity physical activity while the others only exerted mild efforts (i.e., walking). The smartwatch sampling time was significantly smaller than the insulin pump or the CGM sampling time which was 5 min. To obtain a unified sampling time, the heart rate values were averaged over a 5-min time window. Therefore, to test the model’s ability to reproduce the evolution of glycaemia for a given individual, we ran the least-squares identification method offline on *n* samples of the output error.

Our cost function to minimize was thus:

5 $$\begin{aligned} J_n(\theta )=\frac{1}{n} \sum _{k=1}^n(y_{meas}[k]-{\hat{y}}(\theta )[k])^2 \end{aligned}$$

The optimal value $\theta ^{*}$ was the vector of parameters that minimizes the criterion $J_n$. The recursive Gauss-Newton optimization method was initialized with a plausible set of parameters. With the optimal parameter $\theta ^{*}$ comes a minimal criterion $J_n(\theta ^{*})$, a gradient $G_n(\theta ^{*})$ and a Hessian ${\hat{H}}_n(\theta ^{*})$. In practice, the Hessian matrix ${\hat{H}}_n(\theta ^{*})$ appears to be well conditioned. This ensures the practical identifiability. The standard deviation of the parameters estimation error is obtained from the norm of the elements on the main diagonal of the estimated covariance matrix $Var(\theta ^*)$.

6 $$\begin{aligned} Var(\theta ^{*})=\frac{1}{n}J_n(\theta ^{*}){\hat{H}}_n^{-1}(\theta ^{*}) \end{aligned}$$

When calculating the percentage of fit $\%fit$ in the identification procedures, we refered to the relative absolute difference between the real output and estimated output giving the following equation:

7 $$\begin{aligned} \%fit=\frac{\left| Y_{patient}-Y_{identif.} \right| }{Y_{patient}}\times 100 \end{aligned}$$

### The control law

A control law was designed based on the Dynamic Bolus Calculator^19^. This control law is able to self-deliver prandial boluses to patients without any exterior intervention while ensuring the positivity of the control and the system states, and thus preventing hypoglycaemic episodes. A new term was added to adapt insulin delivery to physical activity. In this study, physical activity was considered hypoglycaemic. Thus, in the event of an exertion, the insulin flow rate must be decreased. The new control law is a state-feedback that formalizes and extends the flexible insulin therapy and standard bolus assistants implemented in insulin pumps.

8 $$\begin{aligned} U_{Bol}(t)=k.\left( \frac{G(t)-G_{ref}}{CF}-IOB(t)+\frac{COB(t)}{CIR}-\frac{PA(t)}{PIR}\right) \end{aligned}$$

where $G_{ref}$ is the target reference glycaemia, *IOB*(*t*) in [U] is the insulin-on-board, i.e., the quantity of insulin that is still active from previous injections, which impacts glycaemia. The carbohydrates-on-board, *COB*(*t*) in [g], are defined in the same way and represent the amount of active carbohydrates remaining from previous meals. *CF* is the correction factor and *CIR* is the carbohydrate-to-insulin ratio. *PA*(*t*) is the physical activity variable and *PIR* is the physical activity to insulin ratio expressed in [bpm/U]. The coefficient *k* in $[min^{-1}]$ is a scaling factor that enables the adjustment of the insulin injection delivery speed for security. When facing parameter underestimation issues that could lead to hypoglycaemia, it may be beneficial to adopt a smooth insulin injection profile to assess the decrease in glycaemia and to update the control process accordingly.

By using the notations from the model, (8) can be equivalently restated as:

9 $$\begin{aligned} \tilde{u}_1(t)=k.\left( \frac{\tilde{x}_1(t)}{k_i}-T_i.(\tilde{x}_2(t)+\tilde{x}_3(t))+\frac{k_cT_c}{k_i}.(x_4(t)+x_5(t))-\frac{\beta \tau _{hr}}{k_i}.x_6(t) \right) \end{aligned}$$

The tilde symbol denotes the variation in the variables around their reference values. Therefore, $x_i(t)=\tilde{x}_i(t)+x_{iref}$.

In the event of physical activity, the positivity of the system is no longer guaranteed and it is likely that the glycaemia level will fall below $G_{ref}$, causing the first term to become negative and the so-called *IOB* to become negative as the insulin flows drop below their basal levels. Such quantities lose their physical significance , but all terms are essential for preserving the stability of the control process.

### OHSU simulator

Currently, the UVA-Padova simulator^21^ is the only FDA-approved simulator for validating control algorithms for insulin distribution in type 1 diabetes patients. Unfortunately, this simulator does not include physical activity data and does not model the effects of physical activity on glycaemia. In 2019, Resalat et al. proposed a statistical virtual patient population for the glucoregulatory system in type 1 diabetes patients, including physical activity^15^. Insulin-glucose dynamics were modelled by using the results of Hovorka^22^, and physical activity was accounted for with Hernandez equations^12^. The data of 20 patients with type 1 diabetes obtained under different meals and aerobic activity scenarios were used to generate a virtual patients population. The cohort was then validated by creating virtual twins of the 20 real patients and assessing their relative times spent in the glycaemic target.

This simulator was used to assess the performance of our control law (8) with a two-day scenario including multiple meals and one physical activity period. As Hovorka’s model parameters could not be easily converted into the extended flexible insulin therapy model parameters, we first performed an identification procedure on the scenario to retrieve the parameters allowing us to use our model and our control law on this patients cohort. After that, we ran a simulation for all 99 patients with their glycaemia dynamics according to the simulator equations and the control law being calculated from our model state estimates (4). At each sampling time, the glycaemia state $x_1(t)$ was set to the actual glycaemia value read by the CGM sensor. We finally calculated the time spent in the glycaemic range as well as the time in hypoglycaemia and the time spent in hyperglycaemia.

To present the results graphically, we adapted the control-variability-grid analysis displayed by the UVA-Padova type 1 diabetes patient simulator^23^. Each patient was represented with two coordinates, an X-coordinate related to the minimum blood glucose value reached during the considered time-period, and a Y-coordinate representing the maximum blood glucose value. These values were corrected considering a 95% confidence interval for the blood glucose values. Consequently, different areas of the graph were delineated, each labelled with a letter from A to E, where A was an ideal glycaemic control and E corresponded to a life-threatening scenario in which the patient spent most of their time in hypoglycaemia or in hyperglycaemia.

### Ethics statement

The study was approved by the ethics committee of Nantes Universitary Hospital (ie., the French Committee of Protection of Persons) approval number 2020-058B on 2 February 2021 and was successfully registered at ClinicalTrial.gov with identification number: NCT04572009 (registration date 01/10/2020). All the methods carried out are in accordance with relevant guidelines and regulations. All patients who signed their informed consent forms for participation were included in this prospective study.

## Supplementary Information

Supplementary Information.

## Data Availability

The main data supporting the results in this study are available within the paper and its Supplementary Information. The clinical data came from the NCT04572009 study (https://clinicaltrials.gov/ct2/show/NCT04572009) which is still in progress. Here we share the raw sample data of one patient and we provide the flexible insulin therapy parameters for all patients in the Supplementary Information.

## Code availability

The analysis code is available at https://data.mendeley.com/datasets/x796nk5ysd/draft?a=f30ed41b-ca5a-41d5-8f17-37cd40ff836e. The model, the control law and the identification method have been released under licence CC-BY-4.0.
