# Supplementary material for: A metamodel-based flexible insulin therapy for type 1 diabetes patients subjected to aerobic physical activity
Source: Sci Rep. 2022 May 16;12:8017. doi: 10.1038/s41598-022-11772-x (PMC9110411; doi:10.1038/s41598-022-11772-x)
Supplement: Supplementary file 1 — Supplementary Information. [file 41598_2022_11772_MOESM1_ESM.pdf]

Supplementary Information
A Metamodel-Based Flexible Insulin Therapy for Type 1 Diabetes Patients subjected to Aerobic Physical Activity

Emeric Scharbarg, Joachim Greck, Eric Le Carpentier, Lucy Chaillous and Claude H. Moog

Useful models for the following section are detailed below.
Bergman model [1] adpated by Furler [2] to the case of type 1 diabetes patient:

$$\begin{cases} \dot{G}(t) = -(p_1 + X(t))G(t) + p_1 G_b + \dfrac{D(t)}{V_g} & (1) \\ \dot{X}(t) = -p_2 X(t) + p_3(I(t) - I_b) & (2) \\ \dot{I}(t) = -n(I(t) - I_b) + \dfrac{u(t)}{V_i} & (3) \end{cases}$$

We also remind the glucose effectiveness and insulin sensitivity mathematical definitions introduced by Breton:

$$E = -\frac{\partial \dot{G}}{\partial G} \quad (4)$$

$$S_I = \frac{\partial E_{SS}}{\partial I_{SS}} \quad (5)$$

With E being glucose effectiveness and S_I insulin sensitivity respectively in [min^{-1}] and in [min^{-1}/(µU/ml)]. In an effort to homogenize notations, we will make a slight modification to Furler model by changing $I(t)$ unit to a flow rate in [U/min] and such that glycemia is expressed in [mg/dl].

$$\begin{cases} \dot{G}(t) = -(p_1 + X(t))G(t) + p_1 G_b + \dfrac{D(t)}{V_g} & (6) \\ \dot{X}(t) = -p_2 X(t) + p_3(I(t) - I_b) & (7) \\ \dot{I}(t) = -n(I(t) - I_b) + n_i.u_i(t) & (8) \end{cases}$$

We then have $G(t)$ in [mg/dl], $X(t)$ in [min^{-1}], $I(t)$ in [U/min], $D(t)$ in [g/min] and $u(t)$ in [U/min]. Consequently, the parameters are expressed in the following units: p_1 in [min^{-1}], p_2 in [min^{-1}], p_3 in [min^{-1}/U], V_g in [dl], n and n_i in [min^{-1}]. Insulin sensitivity is thus expressed [U^{-1}].

The new flexible insulin-therapy model will also be used to demonstrate the sensitivity factor's defini-

tions in the following section.

$$\begin{cases} \dot{x}_1(t) = -k_i.x_2(t) + k_c.x_4(t) - \beta.x_6(t) + k_d \\ \dot{x}_2(t) = -\frac{1}{T_i}.x_2(t) + \frac{1}{T_i}.x_3(t) \\ \dot{x}_3(t) = -\frac{1}{T_i}.x_3(t) + \frac{1}{T_i}.u_i(t) \\ \dot{x}_4(t) = -\frac{1}{T_c}.x_4(t) + \frac{1}{T_c}.x_5(t) \\ \dot{x}_5(t) = -\frac{1}{T_c}.x_5(t) + \frac{1}{T_c}.u_c(t) \\ \dot{x}_6(t) = -\frac{1}{\tau_{HR}}.x_6(t) + \frac{1}{\tau_{HR}}.(u_{HR}(t) - HR_b) \end{cases} \tag{9}$$

With x_1 the blood glucose concentration in [mg/dl], x_2 the plasma insulin flow in [U/min], x_3 the insulin flow in the subcutaneous compartment in [U/min], x_4 the carbohydrates flow in the duodenum in [g/min], x_5 the carbohydrates flow in the stomach and x_6 the filtered heart frequency in [bpm]. The model parameters are k_d, the difference between the endogenous hepatic glucose production and the insulin independent glucose consumption which causes the diabetic patient to become hyperglycemic with all other terms being zero, expressed in [mg/dl/min]. The parameter k_i is the insulin sensitivity expressed in [mg/dl/U], k_c is the carbohydrate sensitivity in [/dl], T_i and T_c are time constant in [min] representing respectively the time when the plasma insulin concentration is maximal and the time when the rate of appearance of carbohydrates in blood is at a maximum. Parameter β is the physical activity sensitivity expressed in [mg/dl/bpm/min]. Finally τ_{HR} is a fixed time constant representing the time between the onset of physical activity and the heart rate reaching its steady-state.

Breton's model structure is as follow:

$$\begin{cases} \dot{G} = -p_1(G - G_b) - (1 + \alpha Z)X.G - \beta.Y.G + \frac{D(t)}{V_g} \\ \dot{X} = -p_2 X + p_3(I - I_b) \\ \dot{Y} = -\frac{1}{\tau_{HR}} Y + \frac{1}{\tau_{HR}}(HR - HR_b) \\ \dot{Z} = -(f(Y) + \frac{1}{\tau}).Z + f(Y) \end{cases} \tag{10}$$

State $Y(t)$ expressed in [bpm] is a filtered version of the over-basal heart rate signal $HR(t)$ which is used to quantify the intensity of exertion. State $Z(t)$ is adimensional and is here to express the rapid-on slow off increase in insulin sensitivity caused by exertion above a certain level of activity set in the non-linear function $f(Y)$.

1 Sensitivity of blood glucose concentration to external inputs (insulin, stress, meals, physical activity...)

External inputs are those system variables which are fully independent from other variables. In a mathematical description, those external input variables would just be named after "transcendent variables" since they are not solutions of any predefined equation or differential equation. This is the case for instance of the injected insulin input. It is also the case of external disturbances or of meals. On the opposite, once all external inputs are defined, then internal system variables are deferentially dependent, i.e. they are solutions of some differential equation involving external independent variables.
The results stated in the form of propositions in this section specialize the definitions to specific models, including Bergman's model [1], the Flexible Insulin Therapy model [3], and others.

As suggested by Figure 1.b) of the article, the considered models consist

- in a first order differential equation for the blood glucose concentration G which is subject to external inputs as the injected insulin rate u_i, the ingested carbohydrates u_c, the physical activity HR and eventually other disturbance inputs...
- and in diffusion models of those external inputs.

0-order diffusion models

When the diffusion of external inputs is neglected, and with an abuse of notation, the glycemia dynamics reads as:

$$\dot{G} = \dot{G}(G, u_i, u_c, HR) \tag{11}$$

The following is in order.

- The glucids sensitivity factor GSF is defined as $\partial\dot{G}/\partial u_c$.
- The physical activity sensitivity factor PSF is defined to be $-\partial\dot{G}/\partial HR > 0$.
- The insulin sensitivity factor ISF is $-\partial\dot{G}/\partial u_i > 0$.

All those sensitivity factors are known as *instantaneous gains* or *static gains* of G with respect to the respective inputs.

1st-order diffusion models

When some or all inputs are subject to a 1st-order diffusion dynamics, then the most general model is as follows (abusing again the notation).

$$\left\{\begin{array}{rcl} \dot{G} & = & \dot{G}(G, X_i, X_c, X_{HR}, u_i, u_c, u_{HR}) \\ \dot{X}_i & = & \dot{X}_i(X_i, u_i) \\ \dot{X}_c & = & \dot{X}_c(X_c, u_c) \\ \dot{X}_{HR} & = & \dot{X}_{HR}(X_{HR}, u_{HR}) \end{array}\right. \tag{12}$$

The equation $\dot{X}_i = \dot{X}_i(X_i, u_i)$ models the insulin diffusion compartment, which implies that the insulin on board X_i necessarily vanishes without infusion of external insulin, $u_i = 0$. The mathematical consequence is $\frac{\partial \dot{X}_i}{\partial X_i} < 0$. By the way, $-\frac{\partial \dot{X}_i}{\partial X_i}$ represents nothing but the *inverse of the instantaneous vanishing time constant.* Rewrite the insulin diffusion compartment as $\dot{X}_i = \frac{\partial \dot{X}_i}{\partial X_i} \left(\frac{\partial \dot{X}_i}{\partial X_i}\right)^{-1} \cdot \dot{X}_i(X_i, u_i)$. Thus, the *instantaneous gain* k_i^d *of the insulin diffusion compartment* is $k_i^d = -\left(\frac{\partial \dot{X}_i}{\partial X_i}\right)^{-1} \frac{\partial \dot{X}_i}{\partial u} > 0$. The latter is constant when the dynamics of the insulin diffusion compartment is linear, and then it is equal to the static gain. In the most general case, the static gain is obtained when the instantaneous gain is evaluated at the equilibrium.

At this stage, assume that the functions $\dot{X}_i, \dot{X}_c, \dot{X}_{HR}$ at the right hand side of equations in (12) have a unique equilibrium for a given constant input. For instance, given u_i, the equation $\dot{X}_i(X_i, u_i) = 0$ has one single solution $X_i(u_i)$ (which depends on u_i).

The ISF is then defined as the sum of the gains of all the compartments

$$ISF = -\left[\frac{\partial \dot{G}}{\partial u_i} + \frac{\partial \dot{G}}{\partial X_i}\left(\frac{\partial \dot{X}_i}{\partial X_i}\right)^{-1}\frac{\partial \dot{X}_i}{\partial u_i}\right]. \tag{13}$$

Equation (13) is just the application of the chain rule as the injected insulin lead to the decrease of glycemia through two different compartments. In plain words, the ISF has to take into account not only the immediately active injected insulin u_i, but also X_i representing or related to the so-called 'insulin on board' IoB whose action is delayed due to the diffusion process.

Similar definitions of sensitivity with respect to other inputs and disturbances hold and are based on the gains of the various diffusion compartments of those inputs as the ingested carbohydrates.

A possible comprehensive mathematical characterization of (13) is as follows (up to the sign):

$$ISF = d\dot{G} \cdot \begin{pmatrix} 0 \\ k_i^d \\ 0 \\ 0 \\ 1 \\ 0 \\ 0 \end{pmatrix}$$

where $d\dot{G}$ is the gradient of $\dot{G}$ along vectors $\begin{pmatrix} dG & dX_i & dX_c & dX_{HR} & du_i & du_c & du_{HR} \end{pmatrix}$:

$$d\dot{G} = \begin{pmatrix} \frac{\partial \dot{G}}{\partial G} & \frac{\partial \dot{G}}{\partial X_i} & \frac{\partial \dot{G}}{\partial X_c} & \frac{\partial \dot{G}}{\partial X_{HR}} & \frac{\partial \dot{G}}{\partial u_i} & \frac{\partial \dot{G}}{\partial u_c} & \frac{\partial \dot{G}}{\partial u_{HR}} \end{pmatrix} \tag{14}$$

Very similar definitions for the GSF and the PSF are in order.

2nd-order diffusion models

Assume that the insulin diffusion system constists of the two compartments cascade

$$\begin{cases} \dot{X}_1 &= \dot{X}_1(X_1, X_2, u_i) \\ \dot{X}_2 &= \dot{X}_2(X_2, u_i) \end{cases} \tag{15}$$

and the glycemia dynamics is $\dot{G} = \dot{G}(G, X_1, X_2, X_c, X_{HR}, u_i, u_c, u_{HR})$. Equation (15) can be rewritten as

$$\begin{cases} \dot{X}_1 &= \frac{\partial \dot{X}_1}{\partial X_1} \cdot (\frac{\partial \dot{X}_1}{\partial X_1})^{-1} \cdot \dot{X}_1(X_1, X_2, u_i) \\ \dot{X}_2 &= \frac{\partial \dot{X}_2}{\partial X_2} \cdot (\frac{\partial \dot{X}_2}{\partial X_2})^{-1} \cdot \dot{X}_2(X_2, u_i) \end{cases} \tag{16}$$

The ISF is still the sum of the gains of each compartment and can be computed from (16) as

$$ISF = -\frac{\partial \dot{G}}{\partial u_i} - \frac{\partial \dot{G}}{\partial X_2} \left(\frac{\partial \dot{X}_2}{\partial X_2} \right)^{-1} \frac{\partial \dot{X}_2}{\partial u_i} - \frac{\partial \dot{G}}{\partial X_1} \left(\frac{\partial \dot{X}_1}{\partial X_1} \right)^{-1} \left[\frac{\partial \dot{X}_1}{\partial u_i} + \left(\frac{\partial \dot{X}_2}{\partial X_2} \right)^{-1} \frac{\partial \dot{X}_1}{\partial X_2} \frac{\partial \dot{X}_2}{\partial u_i} \right] \tag{17}$$

Renaming k_{1i}^d the *intantaneous gain* of the diffusion compartment X_1,
$k_{1i}^d = \left(\frac{\partial \dot{X}_1}{\partial X_1} \right)^{-1} \left[\frac{\partial \dot{X}_1}{\partial u_i} + \left(\frac{\partial \dot{X}_2}{\partial X_2} \right)^{-1} \frac{\partial \dot{X}_1}{\partial X_2} \frac{\partial \dot{X}_2}{\partial u_i} \right]$ and k_{2i}^d the *intantaneous gain* of the diffusion compartment X_2, $k_{2i}^d = \left(\frac{\partial \dot{X}_2}{\partial X_2} \right)^{-1} \frac{\partial \dot{X}_2}{\partial u_i}$, the ISF can again be written as the following scalar product

$$ISF = d\dot{G} \cdot \begin{pmatrix} 0 \\ k_{1i}^d \\ k_{2i}^d \\ 0 \\ 0 \\ 1 \\ 0 \\ 0 \end{pmatrix}$$

Higher order diffusion models

The most general model we consider has the form

$$\begin{cases} \dot{G} &= \dot{G}(G, X_i^1, ..., X_i^p, u_i, ..., X_c^1, ..., X_c^q, u_c) \\ \dot{X}_i &= \dot{X}_i(X_i, u_i) \\ &\vdots \\ \dot{X}_c &= \dot{X}_c(X_c, u_c) \end{cases} \tag{18}$$

where several inputs may be involved, though only u_i and u_c are explicited. The insulin diffusion subsystem has order p, whose compartments are X_i^j for $j = 1, ..., p$ and the digestion subsystem consists of q compartments. The notations X_i and X_c stand for $X_i = (X_i^1, ..., X_i^p)$ and $X_c = (X_c^1, ..., X_c^q)$.

Each diffusion subsystem consists of a number of equations equal to the order of the diffusion subsystem. For instance, the insulin subsystem consists of p equations in the p variables $(X_i^1, ..., X_i^p)$.

For clarity, the sensitivity to inputs of (18) is defined as the scalar product of $d\dot{G}$ with the vector of gains of each diffusion compartment $(k_{1i}^d, ..., k_{pi}^d, 1, ..., k_{1c}^d, ..., k_{qc}^d, 1)$. This yields the following definitions as special cases.

$$ISF = d\dot{G} \cdot \begin{pmatrix} 0 \\ k_{1i}^d \\ \vdots \\ k_{pi}^d \\ 1 \\ 0 \\ \vdots \\ 0 \end{pmatrix} \tag{19}$$

$$GSF = d\dot{G} \cdot \begin{pmatrix} 0 \\ 0 \\ \vdots \\ 0 \\ k_{1c}^d \\ \vdots \\ k_{qc}^d \\ 1 \end{pmatrix} \tag{20}$$

and when physical activity is involved, PSF is defined in a very similar vein.

The formal and universal definition for the insulin sensitivity factor

Though there is no formal definition of insulin sensitivity in the literature apart from Bergman specific definition, this notion is used instrumental for both biomathematicians and practioners. The need for a consensual characterization of sensitivity to insulin thus appears obvious. The following definition easily mimics the previous notion of "glucose effectiveness" in [1] and is shown to generalize some existing definitions which are valid for some special cases. Note that the equations (6) (7) and (8) considered here for the minimal model are slightly modified as we changed the plasma insulin unit from a concentration to a flow rate, which is more in line with medical practice and in order to compare the sensitivity factors from different models.

Definition 1 *The insulin sensitivity factor (ISF) is defined as the scalar product between the differential of $\dot{G}$ and vector of gains* $\begin{pmatrix} 0 & k_{1i}^d & \cdots & k_{pi}^d & 1 & 0 & \cdots & 0 \end{pmatrix}^T$.

Proposition 1 *In the special case of the Bergman minimal model (6 - 8) and setting* $u(t) = \frac{n_i}{n}.u_i(t)$*, the insulin sensitivity with respect to* u *equals* $\frac{p_3}{p_2}.G(t)$ *and is expressed in [mg/dl/U].*

PROOF. With the change of variable $u(t) = \frac{n_i}{n}.u_i(t)$ in (8), the plasmatic insulin flow $I(t)$ stabilizes with the injected insulin flow $u(t)$. In the case of the Bergman model, the only insulin-related term intervening in $\dot{G}$ equation is $X(t)$.

Applying above definition:

$$ISF = -\frac{\partial \dot{G}}{\partial X} \cdot \left(\frac{\partial \dot{X}}{\partial X}\right)^{-1} \frac{\partial \dot{X}}{\partial I} \left(\frac{\partial \dot{I}}{\partial I}\right)^{-1} \frac{\partial \dot{I}}{\partial u} = G(t).\frac{p_3}{p_2} \tag{21}$$

The ISF is expressed in [mg/dl/U] which is consistent with the clinical practice but is not a constant value as it is proportional to $G(t)$. ◁

Proposition 2 *In the special case of the Flexible Insulin Therapy model (9), the* ISF *equals* k_i *in [mg/dl/U].*

PROOF. In the FIT model, all the global static gain is reported onto the first dynamics of the blood glucose concentration. It includes a second-order diffusion dynamics for the insulin. Apply Definition 1 to (9):

$$ISF = -\frac{\partial \dot{G}}{\partial x_2} = k_i \tag{22}$$

It is consistent with [3] where the insulin sensitivity factor is expressed in [mg/dl/U] as well. ◁

Proposition 3 *In the special case of Breton's extension of the minimal model (10), the* ISF *equals* $(1 + \alpha.Z(t))\frac{p_3}{p_2}.G(t)$*.*

PROOF. Apply (13) with $\frac{\partial \dot{G}}{\partial X} = -G(1 + \alpha Z)$, $\frac{\partial \dot{X}}{\partial X} = -p_2$ and $\frac{\partial \dot{X}}{\partial I} = p_3$ so that

$$ISF = (1 + \alpha.Z(t))\frac{p_3}{p_2}.G(t).$$

◁

The formal and universal definition for the glucid sensitivity factor

It is definitely natural to define the sensitivity to ingested glucids in a similar vein.

Definition 2 *The glucid sensitivity factor (GSF) is the scalar product between the differential of* $\dot{G}$ *and vector* $\begin{pmatrix}0 & 0 & \cdots & 0 & k_{1c}^d & \cdots & k_{qc}^d & 1 & 0 & \cdots & 0\end{pmatrix}^T$.

Proposition 4 *In the special case of the Bergman model, the* GSF *equals* $\frac{1}{V_g}$*.*

PROOF. This proof is direct as the Bergman model (10) has no additional digestion compartment. We can apply directly the definition valid for 0-order diffusion models.
We have $GSF = \frac{\partial \dot{G}}{\partial D}$ with $\dot{G}(t) = -(p_1 + X(t))G(t) + p_1 G_b + \frac{D(t)}{V_g}$.
Obviously, the GSF equals $\frac{1}{V_g}$ and is expressed in $[dl^{-1}]$. ◁

Proposition 5 *In the special case of the Flexible Insulin Therapy model (9), the* GSF *equals* k_c*.*

PROOF. To prove Proposition 5, we proceed in the same way as for Proposition 2. This time we will focus on the variable x_4 instead of x_2.

$$GSF = -\frac{\partial \dot{G}}{\partial x_4} \cdot 1 = k_c \tag{23}$$

The GSF in this case is still expressed in dl^{-1}. ◁ From a medical point of view, the carbohydrate to insulin ratio CIR is the amount of carbohydrates needed to compensate the injection of 1 unit of insulin.

Definition 3 *The CIR is mathematically defined as the ratio between the insulin sensitivity factor and the glucid sensitivity factor, $CIR = \frac{ISF}{GSF}$.*

Proposition 6 *In the special case of the Bergman model (6 - 8), $CIR = \frac{p_3.V_g}{p_2}.G(t)$.*

PROOF. This follows directly from Propositions 1 and 4. We find out that CIR equals $\frac{p_3.V_g}{p_2}.G(t)$ and is dimensionally homogeneous to [g/U]. ◁

Proposition 7 *In the special case of the FIT model (9), the CIR equals $\frac{k_i}{k_c}$.*

PROOF. This follows directly from Propositions 2 and 5. The CIR for this model equals $\frac{k_i}{k_c}$ and is expressed in [g/U]. ◁

The formal and universal definition for the physical activity sensitivity factor

It is definitely natural to define the sensitivity to physical activity in a similar vein. Assume that the physical activity diffusion system consists of s compartments characterized by their respective gains $k^d_{1p}, ..., k^d_{sp}$.

Definition 4 *The Physical activity Sensitivity Factor (PSF) is the scalar product between the differential of $\dot{G}$ and the column vector $\begin{pmatrix} 0 & 0 & \cdots & 0 & k^d_{1p} & \cdots & k^d_{sp} & 1 \end{pmatrix}^T$.*

Proposition 8 *In the case of the Breton's extension to the minimal model (10), the* instantaneous *PSF equals:*

$$PSF = (\beta + \alpha X)G(t) + \left(\frac{1}{f(Y) + 1/\tau}\right)^{-1} (1 - Z).\frac{df}{dY}$$

PROOF. Model (10) includes a 2-compartments physical activity subsystem. The formula to apply if thus adapted from (17):

$$PSF = -\frac{\partial \dot{G}}{\partial Y} - \frac{\partial \dot{G}}{\partial Z}\left(\frac{\partial \dot{Z}}{\partial Z}\right)^{-1} \frac{\partial \dot{Z}}{\partial Y}.$$

The result follows. ◁

Proposition 9 *In the special case of the extended flexible insulin therapy model (9), the PSF equals β and is expressed in [mg/dl/bpm/min].*

PROOF. The extended FIT model (9) includes a single compartment for the physical activity and formula (13) has to be adapted to the input u_{HR}.
The gain of the x_6 compartment is 1. The PSF is thus expressed as: $PSF = -\frac{\partial \dot{G}}{\partial x_6} = \beta$. ◁

Identifiability and positivity assessment of model and control law

Identifiability

The mathematical characterization of model identifiability reduces to checking if we can derive as many linearly-independent equations as there are parameters θ to be identified. This can be computed as a condition on the rank of the jacobian matrix of successive derivatives of the measured output with respect to the unknowns θ.

$$Rank\left(\frac{\partial(y,\dot{y},...,y^{(i)},...)}{\partial\theta}\right) = m$$

where m is the number of parameters to be identified and where y is the measured output. With complex high order models including nonlinear terms, these computations may become tedious. A first analysis performed on a simplified version of Breton's model suggests that the model is identifiable, except at some singularities where the rank of the jacobian matrix drops, as it is known for generic nonlinear models.

To confirm this intuition, a generating series approach was used to assess structural identifiability of the model using the GenSSI toolbox on MATLAB$^{©}$. This method is a multi-experimental approach where identifiability is assessed with different inputs for the same initial condition. Structural identifiability refers to the ability of a model to remain identifiable for all parameters values almost everywhere. It results that all the parameters of the extended FIT model $\begin{bmatrix} k_d & k_i & T_i & k_c & T_c & \beta \end{bmatrix}^T$ are globally identifiable. This is depicted by the following reduced identifiability table obtained by computing successive Lie derivatives and computing the Jacobian of the generated series along the parameters. A black square means that the corresponding parameter occurs in the series coefficient, otherwise the square remains white:

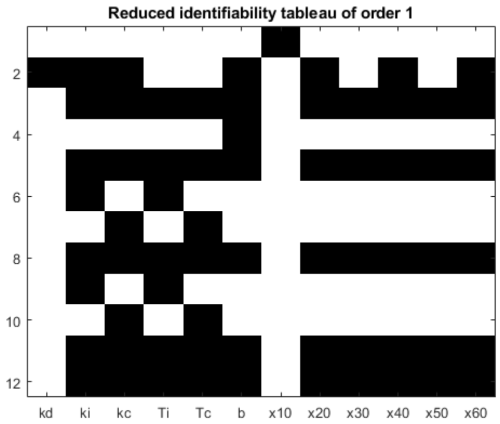

Figure 1: Reduced identifiability table for the new FIT model

Positivity

We consider the following system:

$$\begin{cases} \dot{x}(t) = Ax(t) + Bu(t) \\ y(t) = Cx(t) \end{cases} \tag{24}$$

where $x \in \mathbf{R}^n$, $B \in \mathbf{R}^{n\times m}$ and $C \in \mathbf{R}^{p\times n}$.

Theorem 1 *[4] System (24) is internally positive if and only if:*

1. *A is a Metzler Matrix*
2. *$B \in \mathbf{R}_+^{n\times m}$ and $C \in \mathbf{R}_+^{p\times n}$*

We recall that a matrix is said to be Metzler if its off-diagonal coefficients are positive. Denoting $H = [h_{ij}] \in \mathbf{R}^{q\times l}$, H is Metzler if $h_{ij} \geq 0$ for all $i \neq j$

Our closed-loop systems behaves following the equation:

$$\dot{\tilde{x}}(t) = (A + B_u F_k)\tilde{x}(t) + B_c u_c(t) + B_{hr} u_{hr}(t) \tag{25}$$

We rename $\tilde{A}$ the closed-loop state matrix:

$$\tilde{A} = \begin{pmatrix} 0 & -k_i & 0 & k_c & 0 & -\beta \\ 0 & -\frac{1}{T_i} & \frac{1}{T_i} & 0 & 0 & 0 \\ \frac{k}{k_i T_i} & -k & -\frac{1}{T_i} - k & \frac{k k_c T_c}{k_i T_i} & \frac{k k_c T_c}{k_i T_i} & -\frac{k\beta\tau_{HR}}{k_i T_i} \\ 0 & 0 & 0 & -\frac{1}{T_c} & \frac{1}{T_c} & 0 \\ 0 & 0 & 0 & 0 & -\frac{1}{T_c} & 0 \\ 0 & 0 & 0 & 0 & 0 & -\frac{1}{\tau_{HR}} \end{pmatrix} \tag{26}$$

The closed-loop system is stable as all the eigenvalues are positioned in the left part of the complex plane (a condition is that k must be strictly positive). Controllability of the system is assessed using Kalman's criterion. It shows that three poles are non-controllable by insulin injection. The non-controllable dynamics are found using the Popov-Belevitch-Hautus criterion, in fact, states $\tilde{x}_4$, $\tilde{x}_5$ and $\tilde{x}_6$ are not controllable. If we look at the subsystem made out of these three states, we clearly see that the reduced state matrix A_r is Metzler and reduced input and output matrices, respectively B_r and C_r are positive leading to an internally positive subsystem by application of. Also the carbohydrate input and the physical activity input are both positive. Thus states $\tilde{x}_4$ $\tilde{x}_5$ and $\tilde{x}_6$ are positive.

The following positivity analysis uses results by Farina [4] and Castelan [5] established for autonomous system. We will thus work on the system $\dot{x} = \tilde{A}.x$ based on the above closed-loop state-matrix.

Here we recall a method to determine a positive invariant subset ensuring that all trajectories initialized in this subspace will remain inside of it.

Definition 5 *Let us consider the autonomous system $\dot{x} = Ax$, $x \in \mathbf{R}^n$, and the trajectory $x(t, x_0)$ where x_0 is the initial condition. The set $\Omega \subseteq \mathbf{R}^n$ is positively invariant if $\forall x_0 \in \Omega \Rightarrow x(t, x_0) \in \Omega\, \forall t \geq 0$.*

Given a matrix M, we define $\Omega(M)$ the following polyhedron:

$$\Omega(M) = \{x \in \mathbf{R}^n | Mx \geq 0\} \tag{27}$$

so that $\Omega(M)i$ a subset of $\mathbf{R}^n$.

We will be using a theorem from [5] to find a positive invariant set for our system.

Theorem 2 *The polyhedron $\Omega(M)$ is a positive invariant set (PIS) if and only if there exists a Metzler matrix $H \in \mathbf{R}^{r\times r}$ such that:*

$$MA - HM = 0 \tag{28}$$

In plain words, this theorem considers a state transformation matrix M so that the state matrix $\tilde{A}$ is transformed into the Metzler matrix H. If we pick $M = I_{6\times 6}$, then we arrive that the only solution to (28) is $H = \tilde{A}$ which is not Metzler. This is consistent with the fact that the first quadrant is not a

positive invariant set for this system. We therefore need to reduce the space by adding new equations to the matrix M in order to reduce the polyhedron $\Omega(M)$. The matrix M is a constraint matrix and defines the borders of the polyhedron. New constraints will be added by expanding matrix M with an additional row being the state-feedback equation, as we want both the state-positivity and the control-positivity to be guaranteed. Nevertheless, calculations showed that no solution could be find to 28. An additional generic row is therefore further be added to M, say a row vector parametrized by w_i, $i \in [\![1 : 6]\!]$.

Finally, $M^* = \begin{pmatrix} I_{6\times 6} \\ F_k \\ W \end{pmatrix}$ where $W = \begin{bmatrix} w_1 & w_2 & w_3 & w_4 & w_5 & w_6 \end{bmatrix}$.

In this configuration, we were able to find a Metzler matrix H solution to 28.

$$\begin{pmatrix} k_i & 0 & k_i & k_c + k_i & k_i & 0 & 0 & k_i \\ \frac{1}{T_i} & 0 & \frac{2}{T_i} & \frac{1}{T_i} & \frac{1}{T_i} & \frac{\beta}{k_i T_i} & 0 & \frac{1}{T_i} \\ \frac{1}{T_i} & \frac{1}{T_i} & 0 & \frac{1}{T_i} & \frac{1}{T_i} & \frac{\beta}{k_i T_i} & \frac{1}{T_i} & \frac{1}{T_i} \\ \frac{1}{T_c} & \frac{1}{T_c} & \frac{1}{T_c} & 0 & \frac{2}{T_c} & \frac{\beta}{k_i T_c} & 0 & \frac{1}{T_c} \\ \frac{1}{T_c} & \frac{1}{T_c} & \frac{1}{T_c} & \frac{1}{T_c} & 0 & \frac{\beta}{k_i T_c} & 0 & \frac{1}{T_c} \\ \frac{k_i}{\beta \tau_{hr}} & \frac{k_i}{\beta \tau_{hr}} & \frac{k_i}{\beta \tau_{hr}} & \frac{k_i}{\beta \tau_{hr}} & \frac{k_i}{\beta \tau_{hr}} & 0 & 0 & \frac{k_i}{\beta \tau_{hr}} \\ 0 & 0 & 0 & 0 & 0 & 0 & -k & 0 \\ h_{88} - \frac{k}{k_i T_i} & h_{88} + k_i + \frac{1}{T_i} + k & h_{88} + k & 0 & k_c - \frac{1}{T_c} & h_{88} \frac{\beta}{k_i} + \beta + \beta \frac{k \tau_{hr}}{k_i T_i} + \frac{\beta}{k_i \tau_{hr}} & 0 & h_{88} \end{pmatrix} \tag{29}$$

Pick $h_{88} = k_c + \frac{k k_c T_c}{k_i T_i} - \frac{1}{T_c}$, which is positive with a standard value of system parameters. Vector W is found as follows:

$$W = \begin{bmatrix} -1 & -1 & -1 & -1 & -1 & -\frac{\beta}{k_i} \end{bmatrix} \tag{30}$$

To initialize the system trajectory, the following constraints need to be met:

$$\begin{cases} \tilde{x}_1(0) \geq 0 \\ \tilde{x}_{2,3}(0) \geq 0 \\ \tilde{x}_{4,5,6}(0) \geq 0 \\ \tilde{u}_k(0) \geq 0 \\ W.\tilde{x} \geq 0 \end{cases} \Leftrightarrow \begin{cases} G(0) \geq G_{ref} \\ \exists \tau < 0 | \forall t \geq \tau, u(t) \geq U_b \\ \text{By definition} \\ G(0) - G_{ref} \geq ISF \times (IOB(0) - \frac{COB(0)}{CIR} + \frac{PA(0)}{PIR}) \\ \tilde{x}_1 + \tilde{x}_2 \tilde{x}_3 + \tilde{x}_4 + \tilde{x}_5 + \frac{\beta}{k_i} \tilde{x}_6 \leq 0 \end{cases} \tag{31}$$

The polyhedron found here is not guaranteed to be the largest one as we made some arbitrary assumption, but it is an efficient solution.

2 Parameter identification for our clinical study patient cohort

The following Table 1 presents the identification results from our real patient cohort. As only patient M02 performed significant physical activity, his β parameter evaluated during the identification procedure was used for the 9 other patients to create their virtual twins. More than the numerical results presented in the following table, we want to make sure that the overall dynamics of glycemia is correctly fitted by the estimated model parameters. This fact is assessed with the following Figures 2 to 11. When possible, we found out that running the optimization algorithm on a two days-long data set was providing the best results.

Table 1: Comparison of the patient values of FIT parameters with the identified values and their relative accuracy. The $\hat{}$ variables denote the estimated FIT parameters

**Patient**	**Basal [U/h]**	**ISF [mg/dl/U]**	**CIR [g/U]**	$\widehat{Basal}$	$\widehat{ISF}$	$\widehat{CIR}$
**M01**	1.95	33.3	5.85	1.58±0.28	23.49±14.6	4±1.01
**M02**	0.72	48.75	10	0.74±0.01	45±2.9	12.2±0.33
**M03**	0.79	60	11.5	0.54±0.14	51.7±30	8.4±0.82
**M04**	1.00	50	7.35	1.33±0.04	16.7±1.1	5.93±0.35
**M06**	0.68	80	20	0.53±0.03	47.8±4.46	19.2±2.27
**M07**	0.79	60	11	1.33±0.09	41±6.9	11.4±1.47
**M08**	2.1	40	9.86	1.9±0.03	36.8±5.5	5±0.19
**M09**	1.08	30	7	1.17±0.02	33.9±3.3	6.69±0.24
**M11**	0.77	70	10	0.75±0.02	97±9.27	8.6±0.21
**M12**	0.63	40	3.5	1.24±0.04	18.8±1.4	3.52±0.08

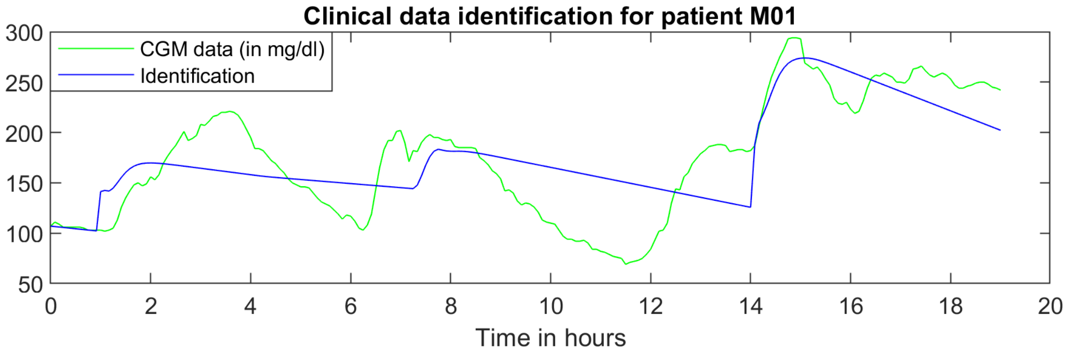

Figure 2: Identification on clinical data for patient M01

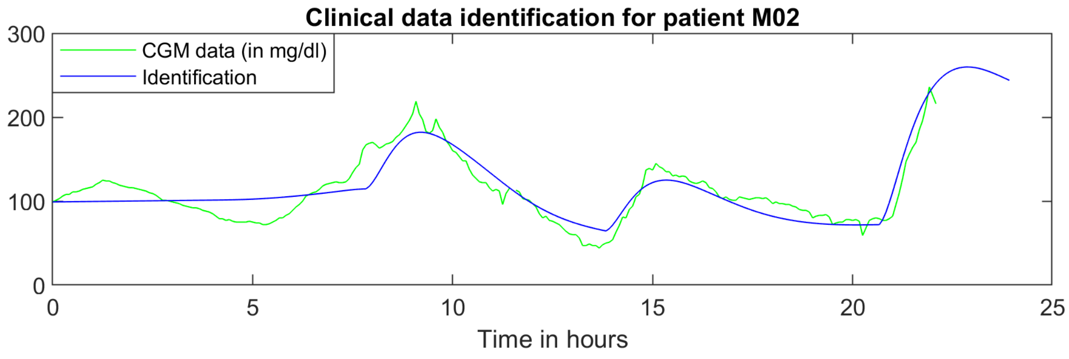

Figure 3: Identification on clinical data for patient M02

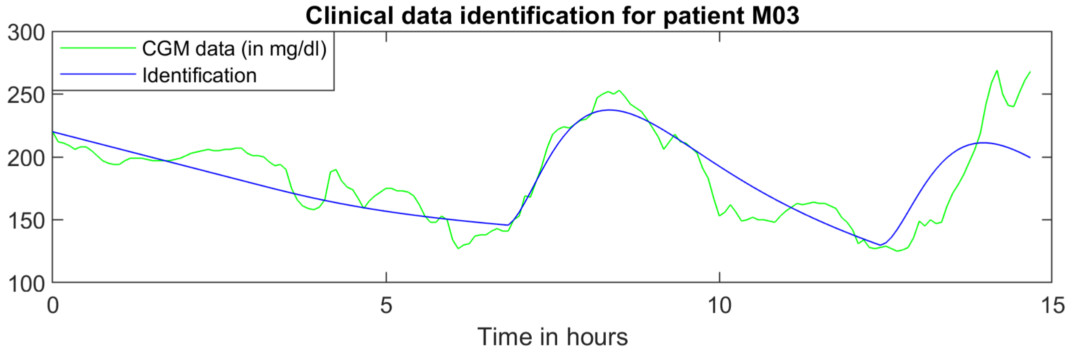

Figure 4: Identification on clinical data for patient M03

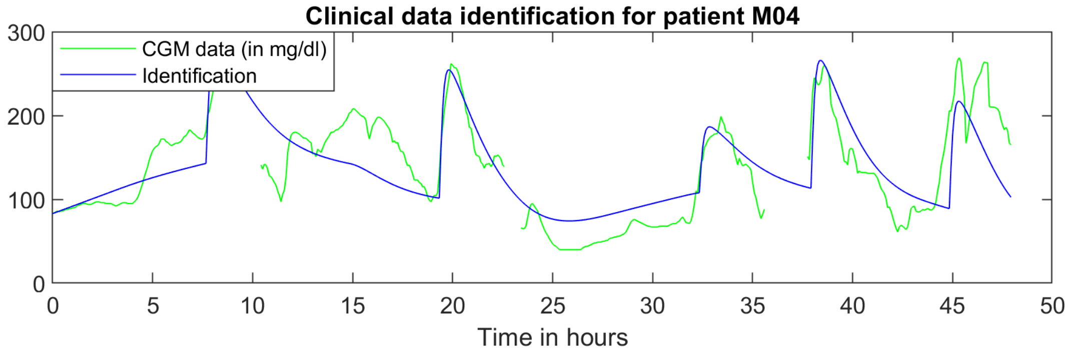

Figure 5: Identification on clinical data for patient M04

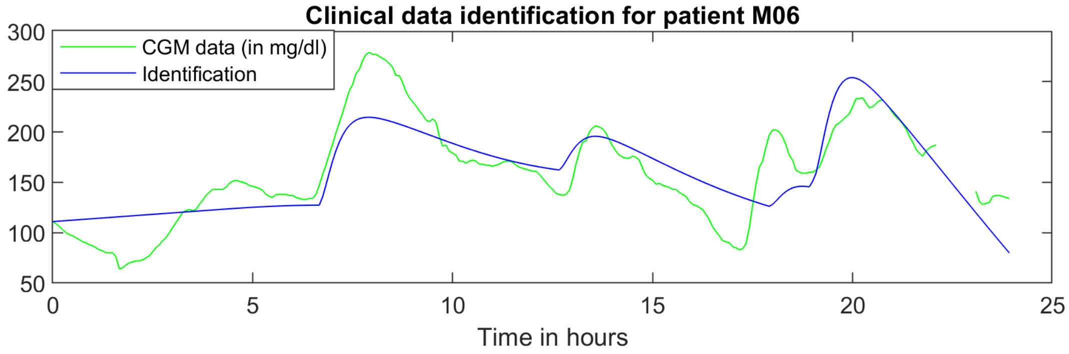

Figure 6: Identification on clinical data for patient M06

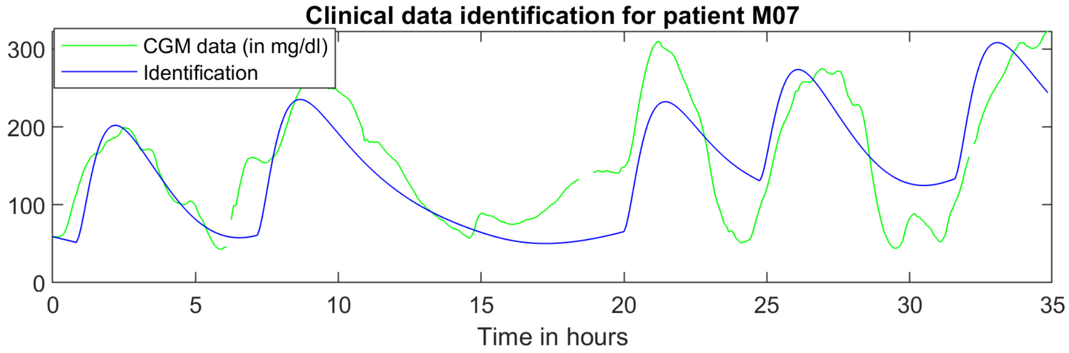

Figure 7: Identification on clinical data for patient M07

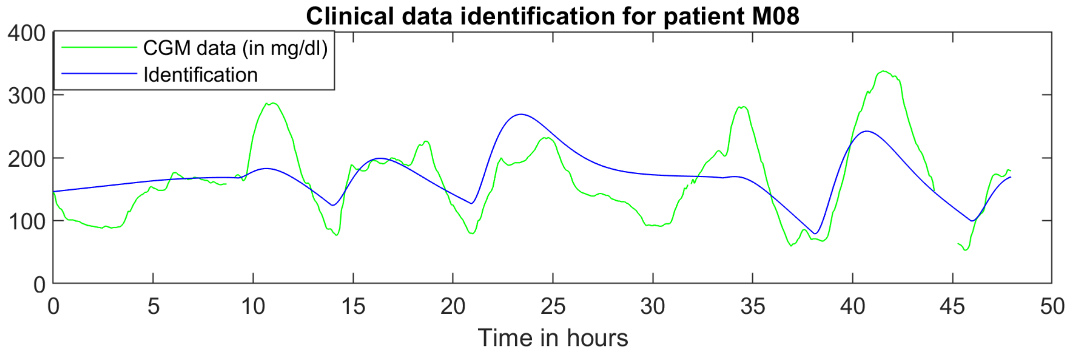

Figure 8: Identification on clinical data for patient M08

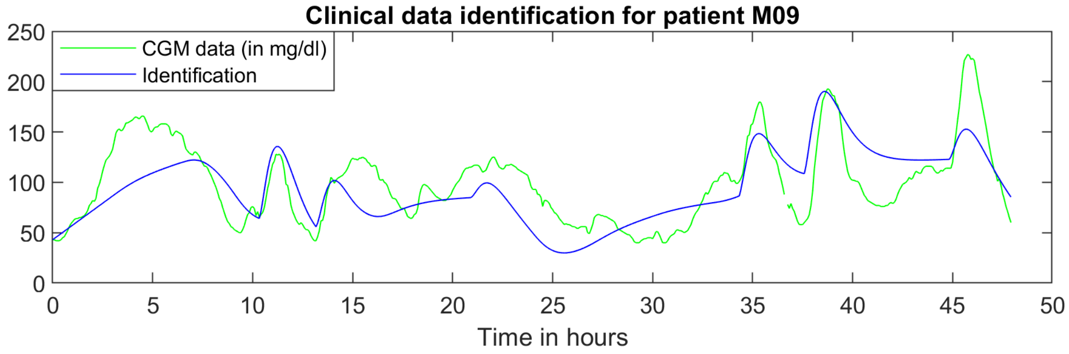

Figure 9: Identification on clinical data for patient M09

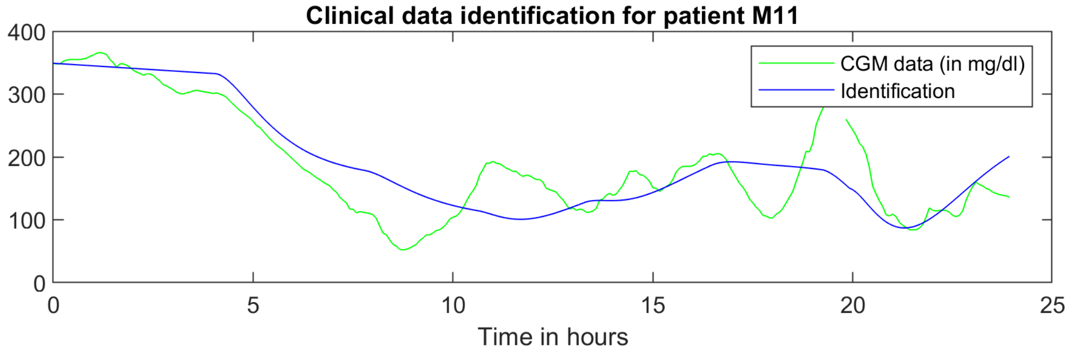

Figure 10: Identification on clinical data for patient M11

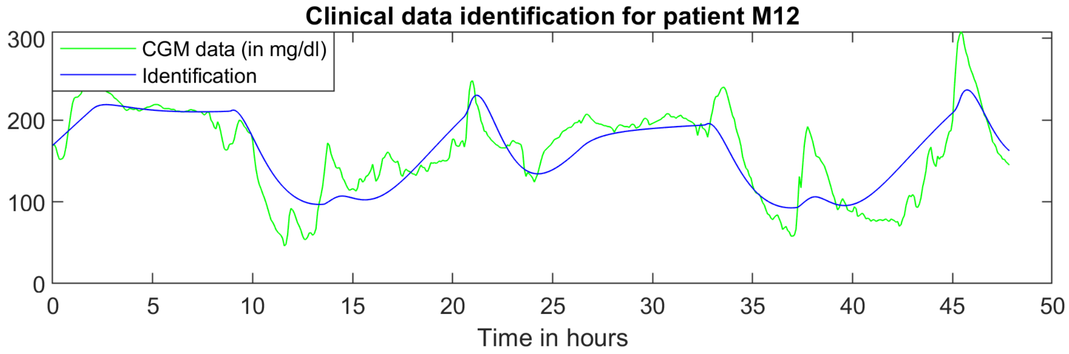

Figure 11: Identification on clinical data for patient M12

References

[1] R.N. Bergman et al. "Quantitative estimation of insulin sensitivity". In: *Am J Physiol.* 233.6 (1979), pp. 667–677. DOI: 10.1152/ajpendo.1979.236.6.E667.

[2] Furler SM et al. "Blood glucose control by intermittent loop closure in the basal mode: computer simulation studies with a diabetic model". In: *Diabetes Care* 8.6 (1985), pp. 553–561. DOI: 10.2337/diacare.8.6.553.

[3] Nicolas Magdelaine et al. "A Long-term Model of the Glucose-Insulin Dynamics of Type 1 Diabetes". In: *IEEE Transactions on Biomedical Engineering* 62.6 (May 2015), pp. 1546–1552. DOI: 10.1109/TBME.2015.2394239. URL: https://hal.archives-ouvertes.fr/hal-01103022.

[4] L. Farina and S. Rinaldi. *Positive Linear Systems: Theory and Applications*. 2000.

[5] E.B. Castelan and Jean-Claude Hennet. "On Invariant Polyhedra of Continuous-time Linear Systems". In: *Automatic Control, IEEE Transactions on* 38 (Dec. 1993), pp. 1680–1685. DOI: 10.1109/9.262058.
